# Pharmacotherapies in Heart Failure With Preserved Ejection Fraction: A Systematic Review and Meta-Analysis of Randomized Controlled Trials

**DOI:** 10.7759/cureus.13604

**Published:** 2021-02-28

**Authors:** Nischit Baral, Swotantra Gautam, Saroj A Yadav, Sangeeta Poudel, Govinda Adhikari, Rohit Rauniyar, Pramod Savarapu, Anjan Katel, Anish C Paudel, Prem R Parajuli

**Affiliations:** 1 Internal Medicine, McLaren Health Care, Flint/Michigan State University, Michigan, USA; 2 General Medicine, B. P. Koirala Institute of Health Sciences, Dharan, NPL; 3 General Medicine, Patan Academy of Health Sciences, Kathmandu, NPL; 4 General Medicine, KIST Medical College/Tribhuvan University, Kathmandu, NPL; 5 Internal Medicine, McLaren Flint/Michigan State University, Flint, USA; 6 Internal Medicine, Kathmandu University School of Medical Sciences, Dhulikhel, NPL; 7 Internal Medicine, Reading Hospital Tower Health, Reading, USA

**Keywords:** pharmacotherapies, heart failure, heart failure with preserved ejection fraction, peak vo2, cardiovascular mortality, hospitalization, all-cause mortality

## Abstract

Background: Heart failure (HF) with preserved ejection fraction (HFpEF) causes significant cardiovascular morbidity and mortality. It is a growing problem in the developed world, especially, in the aging population. There is a paucity of data on the treatment of patients with HFpEF. We aimed to identify pharmacotherapies that improve peak oxygen consumption (peak VO_2_), cardiovascular mortality, and HF hospitalizations in patients with HFpEF.

Methods: We conducted a systematic literature search for English studies in PubMed, EMBASE, Cochrane Central Register of Controlled Trials, Web of Science, Scopus, and Google scholar. We searched databases using terms relating to or describing HFpEF, stage C HFpEF, and diastolic HF and included only randomized controlled trials (RCTs). RevMan 5.4 (The Cochrane Collaboration, 2020, London, UK) was used for data analysis, and two independent investigators performed literature retrieval and data-extraction. We used PRISMA guidelines to report the outcomes. We included 14 articles in our systematic review and six studies in meta-analysis.

Results: We calculated the pooled mean difference (MD) of peak VO_2_ between placebo and pharmacotherapies. Our meta-analysis showed that the peak VO_2_ was comparable between pharmacotherapies and placebo in HFpEF (MD = 0.09, 95% CI: −0.11, 0.30, I^2^ =28%). Our systematic review highlights that statins and spironolactone use should be further studied in larger RCTs due to their potential beneficial effect on all-cause mortality and hospitalizations, respectively.

Conclusion: Compared to placebo, none of the pharmacotherapies significantly improved peak VO2 in HFpEF except ivabradine. In our meta-analysis, the pooled improvement in peak VO_2_ is non-significant. This needs validation with larger studies. We are lacking larger studies on pharmacotherapies that improve peak VO_2_ in HFpEF. Statin and spironolactone should be further studied in patients with HFpEF as few trials have shown improvement in all-cause mortality and reduction in HF hospitalizations in selected patients, respectively.

## Introduction

More than 6.2 million adults in the United States suffer from heart failure (HF) [1]. HF with preserved ejection fraction (HFpEF) composes half of all patients with HF [2]. Patients with HFpEF are more likely to be older, female, and have multiple co-morbid conditions, and no drugs have yet been shown to improve morbidity and mortality [3]. Symptom burden and adverse outcomes of HFpEF are similar to patients with HF with a reduced ejection fraction (HFrEF) [4]. American College of Cardiology/American Heart Association 2017 Guidelines recommend management of HFpEF by treating the contributing factors and comorbidities that are frequently present and significantly impact the clinical course. The most common include hypertension, lung disease, coronary artery disease, obesity, anemia, diabetes mellitus, kidney disease, and sleep-disordered breathing [5]. There is a paucity of data on newer pharmacotherapies in HFpEF. The aim of this analysis was to identify pharmacotherapies that improve peak oxygen consumption (peak VO_2_), cardiovascular mortality, and HF hospitalizations in patients with HFpEF.

## Materials and methods

Search strategy

A comprehensive literature search was performed on PubMed, Cochrane database, Embase, Google Scholar, and Web of Science identifying using relevant Medical Subject Headings (MeSH) and key word termed HFpEF (Heart Failure with Preserved Ejection Fraction) or HFnEF (Heart Failure with Normal Ejection Fraction) and “management,” “pharmacotherapy,” “future therapy,” “Neprilysin inhibitor,” “sacubitril,” “valsaltran,” “Interleukin-1 Blocker,” “anakinra,” “Phosphodiesterase-5 inhibitor,” “sildenafil,” “If-channel inhibitor,” “Ivabradine,” “endothelin type A receptor antagonist,” “sitaxsentan,” “inhaled β-adrenergic agonist,” “albuterol,” “metformin,” “luseogliflozin,” “voglibose,” “Ranolazine,” “statins,” “digoxin,” “Neladenoson,” “Erythropoietin,” “Epo,” “L-arginine L-citrulline,” “Serelaxin,” “Spironolactone,” “aldosterone antagonist,” and “CoQ” with additional filters of human studies and customized articles in accordance with Preferred Reporting Items for Systematic Reviews and Meta‐analysis (PRISMA) guidelines [6]. A staged literature search was performed. All identified articles reference lists were analyzed for additional studies through further snowball sampling. All relevant articles were screened and only appropriate articles included after full-text analysis.

Inclusion and exclusion criteria

We included human studies on patients with diagnosed HFpEF based on an ejection fraction more than or equal to 45% and discussing management of HFpEF for full-text analysis. We excluded editorials, consensus documents, commentaries, review articles, and case reports. We excluded studies with an ejection fraction less than 45%.

Data extraction

All articles were screened by two authors and any disagreement was reached by consensus or involvement of a third author. Data were extracted by two authors and validated by a third author.

Risk of Bias Assessment

**Figure 1:**
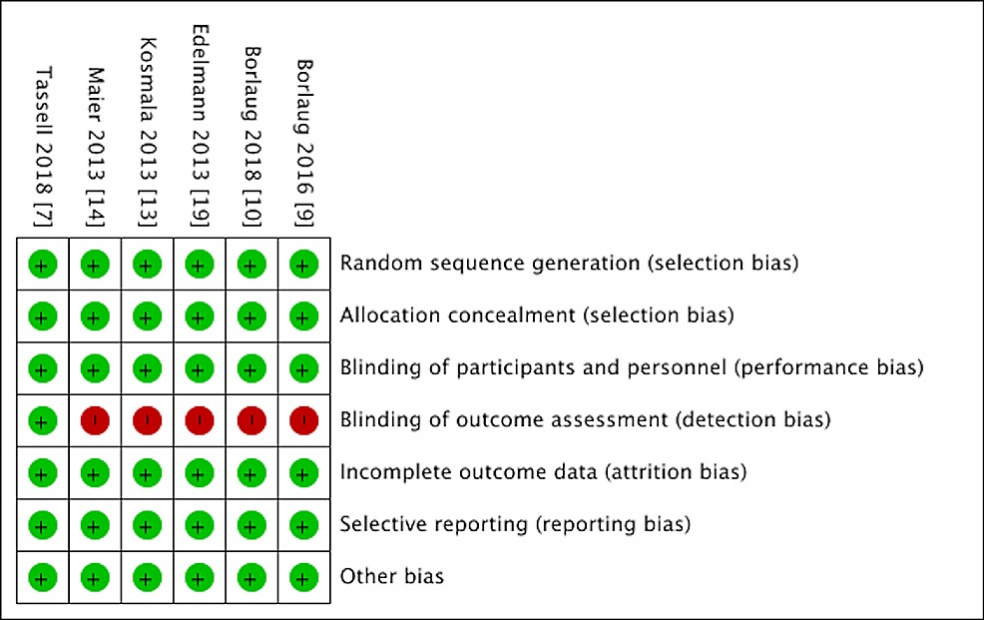
Cochrane Risk of Bias tool showing the risk of bias in included randomized controlled trials

Cochrane Collaboration risk of bias tool was used to assess the risk of bias. The quality of included studies was assessed by two authors with the help of the Cochrane Risk of Bias assessment tool. The risk of bias of the included studies was graded as low in the following aspects: random sequence generation, allocation concealment, blinding of participants and personnel, incomplete outcome data, selective reporting, and other biases. The risk of bias in the blinding of outcome assessment was graded as high (Figure 1).

## Results

Studies included

**Figure 2:**
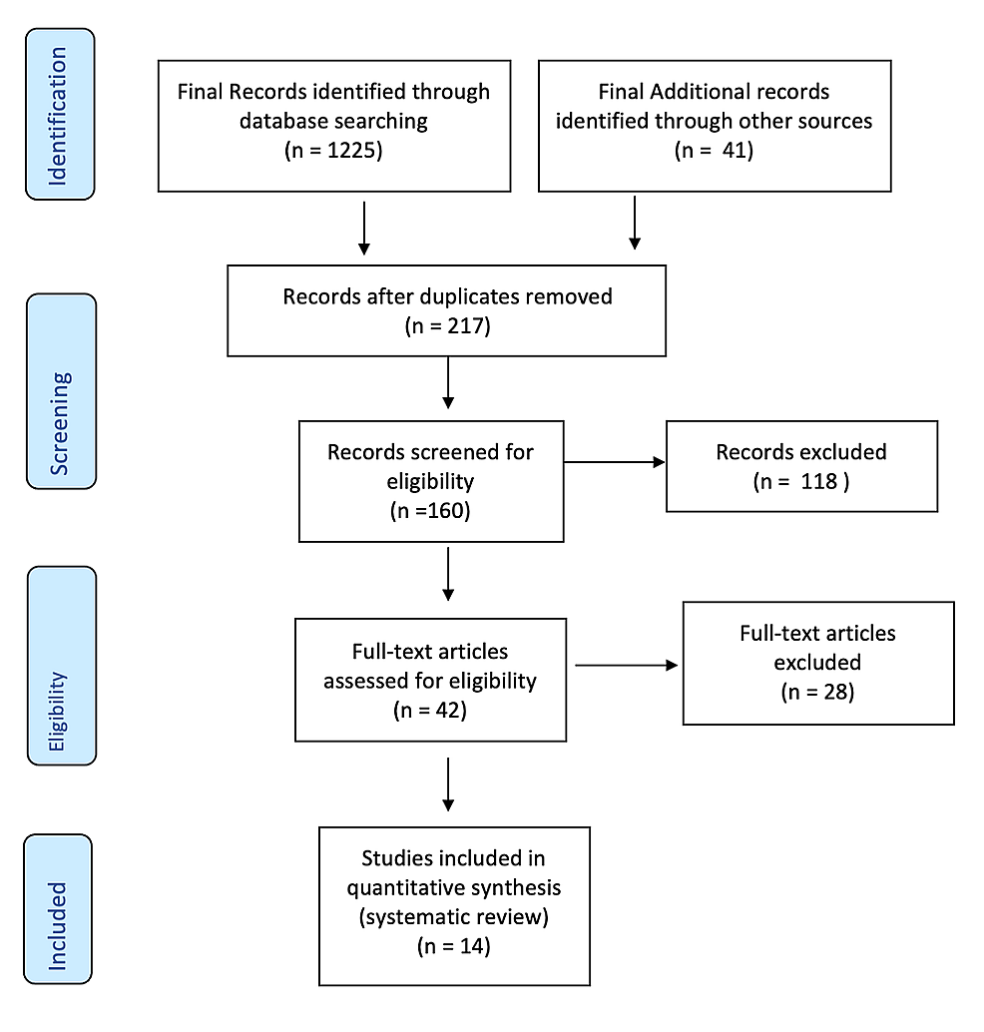
PRISMA flow diagram of included studies PRISMA: preferred reporting items for systematic reviews and meta-analysis

The search using the appropriate terms in January 2021 yielded 1225 potentially relevant articles. In addition, 41 potential articles were included through Web of Science, Embase.com, and Review of references. We included only 14 articles randomizing 6370 participates for 10 different pharmacotherapies according to the homogeneity of these studies with our inclusion criteria (Figure 2).

Outcomes

Characteristics of Included Studies

We included six of the RCTs commenting on peak VO_2_ for the meta-analysis comparing pharmacotherapies with placebo. The rest of the RCTs did not comment on peak VO_^2^_ (Table 1).

**Table 1:** Characteristics of Included Studies 6MWD: six-minute walk distance, peak VO_2_: peak oxygen consumption, QoL: quality of life, NYHA: New York Heart Association, LVEF: left ventricular ejection fraction, RCT: randomized controlled trial, N: number of participants, HFpEF: heart failure with preserved ejection fraction, eGFR: estimated glomerular filtration rate, PCWP: pulmonary capillary wedge pressure, LVEDP: left ventricular end-diastolic pressure, DIG: digitalis investigation group, DHART-2: diastolic heart failure Anakinra response trial 2, CHART-2: congestive heart failure cardiopoietic regenerative therapy, RALI-DHF: Ranolazine in diastolic heart failure, ALDO-DHF: aldosterone receptor blockade in diastolic heart failure, RELAX-AHF: Relaxin for the treatment of acute heart failure, TOPCAT: aldosterone antagonist therapy for adults with heart failure and preserved systolic.

S.N.	Study drug and trial	Type of study	Inclusion criteria	Sample size	Outcome: Improvement seen in		
					Mortality benefits	Hemodynamics and biomarkers	Changes in peak VO_2_, 6MWD, QoL
1	Anakinra DHART 2 trial [7]	Double-blind, placebo-controlled RCT	LVEF≥50% NYHA class II-III	N=31	-	Reduction in CRP and NT-proBNP	No improvement is seen. No significant improvement in peak VO_2_.
2	Sildenafil RELAX trial [8]	Multicenter, double-blind, parallel-group RCT	LVEF≥50%	N=216	-	-	No improvement was seen in peak VO_2_ and 6MWD.
3	Nebulized inhaled sodium nitrite [9]	Single-center, double-blind, parallel-group RCT	LVEF≥50%	N=26	Not available	No any improvement in CO or stroke volume	Reduces PCWP, biventricular filling pressure, and pulmonary artery pressure.
4	Inorganic nitrite INDIE-HFpEF trial [10]	Multicenter, double-blind, placebo-controlled, 2-treatment, crossover trial	LVEF≥50%	N=105	-	-	No improvement was seen in peak VO_2_ after treatment for four weeks.
5	Statin (CHART-2) [11]	An observational study from Japanese registry	LVEF≥50%	N=4544	Reduced incidence of all-cause death, non-cardiovascular death, and sudden death.		Not measured in the trial
6	Digoxin DIG trial [12]	Subgroup and retrospective analysis from DIG trial	LVEF≥50%	N=719	No mortality benefit in the subgroup of HFpEF.		No statistically significant reduction in hospitalization in the HFpEF subgroup.
7	Ivabradine [13]		LVEF≥50%	N=61	-	LV filling pressure	A significant change in exercise capacity and peak VO_2_.
8	Ranolazine RALI-DHF trial [14]	Prospective, double-blind, placebo-controlled RCT	LVEF≥45%	N=20	-	Decrease LVEDP and PCWP	No significant change in peak VO_2_ after 14 days of Ranolazine.
9	Sitaxsentan [15]	Multicenter, double-blind, RCT	LVEF≥50%, NHYA class II-III	N=192	-	-	Improvement in treadmill exercise time after six months and exercise tolerance
10	Serelaxin (RELAX-AHF) [16]	RCT, multicenter, double-blind, placebo-controlled	LVEF≥50%	N=281	No mortality benefits	-	Improved dyspnea
12	Sacubitril–valsartan PARAGON-HF trial [17]	Prospective, multicenter, double-blind, RCT	LVEF≥45%, comparison of ARNI (Sacubitril-Valsartan) versus ARB (Valsartan), NYHA II-IV	N=4822	No mortality benefit and not significantly lower rate of total HFpEF hospitalizations.	-	No significant change in the quality-of-life score.
13	Spironolactone TOPCAT trial [18]	International, multicenter, double-blind RCT	LVEF≥45%, stage C HFpEF, hospitalization within 12 months or elevated BNP/NTpro-BNP. Exclusion: uncontrolled HTN, serum potassium > 5.0 mmol/L, creatinine >2.5 mg/dl, or eGFR <30 mL/min per 1.73 m^2^.	N=3445	No change in the primary composite outcome event (cardiovascular mortality, aborted cardiac arrest, or hospitalizations for HF) rate. The only reduction in the HF hospitalization rate in the treatment group.	-	Did not comment on peak VO_2_ or quality of life.
14	Spironolactone ALDO-DHF trial [19]	Prospective, multicenter, double-blind RCT	LVEF≥50%, NYHA class II-III	N=422	No change in hospitalizations.	Modestly increased serum potassium and decreased eGFR.	No change in peak VO_2_ and quality of life. Slightly reduced 6MWD.

**Figure 3:**
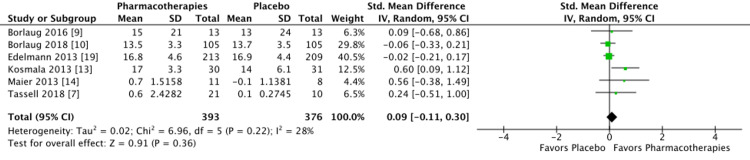
Forest plot showing a change in peak oxygen consumption between pharmacotherapies versus placebo

Change in Peak VO_2_ among the RCTs: the pooled results from six studies showed that the mean difference in peak VO_2_ between pharmacotherapies versus placebo was 0.09, 95% CI: −0.11, 0.30, I2 =28%. This shows that the mean difference of peak VO_2_ between the two groups is comparable (Figure 3).

## Discussion

Pharmacotherapies showing improvement of peak VO_2_

In our study, the pooled increase in peak VO_2_ of 0.09 ml/kg/min is not statistically significant. Peak VO_2_ is an objective parameter for cardiorespiratory fitness. In a study by Mancini et al., the increase in peak VO_2_ from 10 ml/kg/min to 14 ml/kg/min in HF patients was associated with a high increase in cumulative survival [20]. In a recent study on inspiratory muscle training in HFpEF, inspiratory muscle training was associated with an increase in peak VO_2_ and six minutes walk distance [21]. In our study, the change in peak VO_2_ with pharmacotherapies is comparable with placebo.

Pharmacotherapies showing a mortality benefit

Out of the 14 included RCTs, only six RCTs or post-hoc of RCTs compared the all-cause mortality with placebo in HFpEF. In CHART-2 trial, the incidence of three-year mortality was lower in statin group compared to placebo (8.7% vs 14.5%, HR: 0.74; 95% CI; 0.58, 0.94) [11]. In the DIG trial, there was a total of 87 deaths in the digoxin group and 89 deaths in the placebo (HR: 1.06; 95% CI; 0.79, 1.42) [12]. In RELAX-AHF trial, there were total 11 (8.08%) deaths in serelaxin group and 16 (11.32%) deaths in placebo group (HR: 0.70; 95% CI;0.32, 1.50) [16]. In the TOPCAT trial, the primary composite event (cardiovascular death, aborted cardiac arrest, or hospitalizations for HF) rate was not significantly reduced. However, only the hospitalization for HF had a statistically significant reduction in the treatment group compared to placebo (HR 0.83; 95% CI; 0.69-0.99) [18,22]. It is clearly evident from the trials that there have been no promising results for mortality benefit or hospitalization except with statin and spironolactone in selected patients with HFpEF (with EF ≥45%, elevated BNP or HF admission within one year, estimated glomerular filtration rate >30 and creatinine <2.5 mg/dl, potassium <5.0 mEq /L), to decrease hospitalizations patients [22]. However, no improvement was seen in the quality of life with statin [11]. Another study by Alehagen et al. done from prospective Swedish Heart Failure Registry in 9140 with HFpEF with EF more than or equal to 50%, 3427 patients were treated with a statin. Statin showed benefits by reducing cardiovascular death (HR: 0.80; 95% CI; 0.72-0.89; P<0.001) and composite all-cause mortality or cardiovascular hospitalizations (HR: 0.89; 95% CI; 0.82-0.96; P=0.0003) [23]. 

Pharmacotherapies showing improvement in hemodynamics

The study by Kosmala et al. showed improved LV filling pressure and improvement in exercise capacity (metabolic equivalent) when treated with Ivabradine, a selective sinus node inward “funny” (If) channel inhibitor. The study measured these markers only at rest, not during exercise, and the sample size was only 61 [13]. In the RALI-DHF trial with 20 participants, Ranolazine decreased LV end-diastolic pressure and pulmonary capillary wedge pressure [14]. The study by Borlaug et al. showed inhaled sodium nitrite reduces biventricular filling pressures and pulmonary artery pressures at rest and during exercise in HFpEF [9]. In elderly patients with HFpEF, oral nitrate (delivered as beetroot juice) improves exercise capacity, vasodilation, and cardiac output reserve. This study shows inhaled nitrite could be of potential use for exercise and quality of life improvement for HFpEF [24].

Limitations

In our systematic review and meta-analysis, we found a limited number of studies done on novel pharmacotherapies and our sample size is not large enough to provide sufficient power. Definitions for HFpEF were not standardized. Ten of the 12 studies defined an EF of ≥50% as HFpEF, while two of the RCTs defined an EF of ≥45% as HFpEF. This varied cutoff used in RCTs to define HFpEF shows a lack of a universal approach in defining HFpEF [5].

## Conclusions

The mortality, morbidity, and economic burden of HFpEF are huge. There are no clear-cut interventions to the date shown to have mortality benefits in such patients. Uniform definitions for the disease and a consensus on disease management are lacking. Many new pathophysiological models seem to be promising and can be potential targets for the future. Compared to placebo, none of the pharmacotherapies improved peak VO_2_ in HFpEF except ivabradine. This needs validation with larger studies. Statin and spironolactone should be further studied in patients with HFpEF as few trials have shown improvement in all-cause mortality and reduction in HF hospitalizations in selected patients, respectively.
